# Ketogenic therapy as a potential novel treatment approach for PTSD: an integrative review and proposed mechanistic model

**DOI:** 10.3389/fpsyt.2026.1854499

**Published:** 2026-09-09

**Authors:** Robin Engelhardt, John O. Ogedengbe, Malvina S. Millet, Mira E. G. Ayad, Inga Schalinski, Timo Von Oertzen, Anna Rieckmann, David Effinger, Simon Hirschberger, Christopher M. Palmer, Georgia Ede, Shane W. Kraus, Dominik Trommer, Louis Schreel, Julia Tulipan, Bailey Way, Roman-Ulrich Müller, Allison Hull, Jürgen Maes, Marc N. Potenza

**Affiliations:** 1 Institute for Psychology, Faculty of Human Sciences, Universität der Bundeswehr München, Munich, Germany; 2 Thomas Bayes Institute, Berlin, Germany; 3 Department of Human Physiology, Faculty of Basic Medical Sciences, University of Abuja, Abuja, Nigeria; 4 Division of Depression and Anxiety, McLean Hospital, Department of Psychiatry, Harvard Medical School, Cambridge, MA, United States; 5 Department of Psychiatry, Harvard Medical School, Boston, MA, United States; 6 Metabolic and Mental Health Program, McLean Hospital, Harvard Medical School, Belmont, MA, United States; 7 Institut de Génomique Fonctionnelle (IGF), University of Montpellier, CNRS, INSERM, Montpellier, France; 8 Kasr Al Ainy Medical School, Cairo University, Cairo, Egypt; 9 Hochschulambulanz der Universität der Bundeswehr München, Munich, Germany; 10 Walter Brendel Center of Experimental Medicine, Ludwig-Maximilian-University, Munich, Germany; 11 Department of Anesthesiology, Research Unit Immune Function and Immune Metabolism, LMU University Hospital, Munich, Germany; 12 Georgia Ede MD PLLC, Newburyport, MA, United States; 13 Department of Psychiatry, UNLV School of Medicine, University of Nevada, Las Vegas, Las Vegas, NV, United States; 14 Department of Psychology, University of Nevada, Las Vegas, Las Vegas, NV, United States; 15 Department of Philosophy and Moral Sciences, Ghent University, Gand, Belgium; 16 Independent Researcher, Vienna, Austria; 17 Department of Nephrology, Medical Faculty, University Hospital Düsseldorf, Heinrich-Heine-University Düsseldorf, Düsseldorf, Germany; 18 Center for Rare Diseases Cologne, Faculty of Medicine and University Hospital Cologne, Cologne, Germany; 19 Center of Excellence in Nephrology, University Hospitals Aachen, Cologne, Dusseldorf, North Rhine-Westphalia, Germany; 20 Cologne Excellence Cluster on Cellular Stress Responses in Aging-Associated Diseases (CECAD), University of Cologne and University Hospital Cologne, Cologne, Germany; 21 Department of Internal Medicine and Pediatrics, FMC/Orlando Health, University of South Florida, Tampa, FL, United States; 22 The R3 Institute, Tampa, FL, United States; 23 Department of Psychiatry and Child Study Center, Yale University School of Medicine, New Haven, CT, United States; 24 Department of Neuroscience and Wu Tsai Institute, Yale University, New Haven, CT, United States; 25 Connecticut Mental Health Center, New Haven, CT, United States; 26 Connecticut Council on Problem Gambling, Hartford, CT, United States

**Keywords:** keto, ketogenic, metabolic, metabolism, PTSD, posttraumatic stress disorder, treatment

## Abstract

**Background:**

Brain and body metabolic disruptions have been suggested in several studies as a mechanism underlying posttraumatic stress disorder (PTSD). Therapies targeting metabolic pathways might provide new treatment options. This review explores the potential of ketogenic therapies, such as the ketogenic diet and ketone supplementation, as novel metabolic treatments for PTSD.

**Methods:**

An integrative review was conducted. We searched PubMed, Scopus, PsycINFO, PsycArticles, PSYNDEX, MEDLINE, ClinicalTrials.gov, International Clinical Trials Registry Platform, and EU Clinical Trials Register for clinical, preclinical, observational, mechanistic, and review studies that reported outcomes or mechanisms on the intersection of ketogenic therapy and PTSD. Methodological quality of included studies was assessed independently by two reviewers based on predefined criteria.

**Results:**

Sixteen articles (9 human, 4 animal studies, 3 reviews) were eligible and included in the present review. Several metabolic and signaling pathways through which ketogenic therapies could impact PTSD pathophysiology were identified. Preclinical data suggestive of potential efficacy were identified, while only minimal reliable information is available from human studies.

**Discussion:**

Importantly, the absence of larger clinical trials and the reliance on low-level evidence substantially limit current interpretations. High-quality randomized and adequately powered clinical trials are required to examine the potential role of ketogenic therapies in treating PTSD.

## Highlights

- Emerging findings suggest ketogenic therapy might be beneficial for PTSD.
- Ketone signaling and metabolic pathways may impact PTSD pathophysiology.
- No PTSD-specific feasibility or safety signals for ketogenic therapy were reported.

## 1 Introduction

Post-traumatic stress disorder (PTSD) is a psychiatric condition that can develop after the experience of one or more traumatic events such as actual or threatened death, serious injury, or sexual violence. According to the *Diagnostic and Statistical Manual of Mental Disorders, Fifth Edition, Text Revision* (DSM-5-TR), PTSD is characterized by symptoms such as re-experiencing trauma, avoidance of trauma-related stimuli, enduring alterations in cognition and mood, heightened arousal, and significant distress and/or functional impairment (1). Epidemiological data suggest a cross-national PTSD lifetime prevalence of 3.9% in the general population and of 5.6% among trauma-exposed individuals (2), with considerably higher prevalence rates typically reported in war-affected populations (3).

### 1.1 Etiology, current treatments, and challenges

PTSD is widely understood as a multifactorial disorder arising from an interplay of environmental, psychological, and biological factors (4). From the environmental perspective, in most individuals with PTSD, symptoms arise not from a single incident but from the cumulative impact of multiple, often interacting traumatic experiences across the lifespan. Such cumulative exposure may involve diverse domains, including natural disasters (e.g., earthquakes), accidents (e.g., traffic or maritime incidents), interpersonal violence (e.g., torture, war-related trauma, childhood abuse and neglect), risks associated with displacement and human trafficking, as well as repeated exposure to threat among operational personnel (e.g., 5–7).

Psychological models emphasize trauma-based mechanisms (8, 9), while recognizing individual variability in response to trauma (9, 10). Neurobiologically, trauma-induced structural and functional alterations in the brain, and the dysregulation of the hypothalamic-pituitary-adrenal (HPA) axis alongside neuro-immunological alterations have been associated with PTSD (11–13). Classic psychiatric theories emphasize neurotransmitter dysregulation, highlighting the comprehensive involvement of diverse transmitter systems including serotonin, epinephrine, glutamate, and gamma-aminobutyric acid (GABA; 14). Despite advances in psychopharmacology, medications targeting these transmitter systems have shown limited efficacy in treating PTSD, with remission rates of only 20-30% (14, 15).

Trauma-focused psychotherapy remains a gold standard treatment for PTSD (16–18). However, psychotherapies face barriers, including a shortage of specialized therapists, significant time commitments, high attrition rates, financial constraints, and insurance-related barriers (12, 19). Access to effective treatment is especially constrained in conflict/post-conflict populations, where PTSD prevalence is highest (20). Refugees of war face additional obstacles, such as cultural and language barriers (21, 22), that may limit access to trauma therapy, even in Western countries with robust mental health infrastructures. These limitations underscore the need for exploring novel approaches to enhance current PTSD treatments.

### 1.2 The role of metabolism

Emerging research suggests metabolic disruptions in the brain as a pathway underlying diverse psychiatric conditions, including PTSD (11, 23–26). For instance, trauma-induced alterations in the HPA axis appear to negatively impact metabolic functioning (e.g., chronic serum glucose and immune alterations; 11). Relatedly, metabolic syndrome and alterations in metabolic markers (e.g., insulin resistance, fasting glucose) are commonly observed in individuals with PTSD (reviewed in 25).

Other biological changes associated with PTSD are increased oxidative stress (11) and elevated inflammatory markers (e.g., inflammatory cytokines; 25). Inflammation may contribute to the vulnerability of an individual to develop PTSD after trauma exposure (25) and has been posited as a mechanism underlying PTSD (11). As suggested by Mellon et al. (25) and Palmer (26), mitochondrial dysfunction may link metabolic abnormalities and inflammation. In fact, pathway analysis of a large, multi-omic database of individuals with PTSD and major depressive disorder found the most common disease-associated pathways related to immune mechanisms, metabolism, mitochondrial function, neuronal or synaptic regulation, and stress hormone signaling (27). Recognizing these connections, both Michopoulos et al. (11) and Mellon et al. (25) advocate exploring novel treatments for PTSD by directly targeting metabolism.

### 1.3 Ketogenic therapy: a promising treatment?

Given the growing recognition of metabolic disruptions in PTSD, targeting metabolic pathways presents a promising avenue for treatment. One such treatment option is ketogenic therapy (KT), a set of therapeutic interventions resulting in increased serum ketone levels. KT is most commonly implemented by the ketogenic diet. The ketogenic diet is a high-fat, very low-carbohydrate diet that induces ketogenesis, shifting the body’s and brain’s energy source progressively from glucose to ketones (28). More precisely, limited carbohydrate uptake leads to utilization of body fat, inducing the hepatic synthesis of ketone bodies, primarily β-hydroxybutyrate. Ketone bodies provide a potent supplemental source of energy to extrahepatic tissues by their production of adenosine triphosphate (ATP) via mitochondrial oxidative phosphorylation (29).

Originally developed as a treatment for epilepsy in the 1920s, the ketogenic diet has been extensively studied for its anti-seizure effects (30–32). Proposed mechanisms underlying these effects include improvement in brain metabolism (e.g., enhanced ATP production), reduced inflammation, and altered regulation of neurotransmitter signaling (32). Notably, many of these mechanisms appear to be inverse of those implicated in the pathogenesis of PTSD (11, 14, 25, 26). Moreover, the ketogenic diet seems to impact apoptosis and reduces oxidative stress (23, 24, 32). Both cellular processes appear relevant to mental health (23, 24) and are closely associated with mitochondrial functioning (24, 33, 34) and PTSD pathophysiology (11, 35, 36).

Other forms of KT include supplementation with exogenous ketones through ketone salts, esters, free ketone supplements (37–39), or the supplementation of ketone precursors like medium-chain triglycerides (MCTs; 40, 41), aiming to elevate serum ketone concentrations. Preliminary evidence, including preclinical and pilot studies, suggests that KT might be an effective treatment or treatment addition for various mental disorders (reviewed in 23). Early-stage efficacy has been observed across conditions such as schizophrenia (42–48), bipolar disorder (48, 49), major depressive disorder (50), anorexia nervosa (51, 52), and schizoaffective disorder (53). Given the limitations of current treatments and potential transdiagnostic metabolic pathways in mental disorders, the effectiveness of KT as a metabolic treatment for PTSD warrants timely investigation.

### 1.4 Current study

The primary aim of the present integrative review was to explore the potential of KT as a treatment for PTSD. We achieved this aim by examining the existing body of research on the intersection between KT and PTSD. While primarily focusing on efficacy and mechanistic evidence, this study’s secondary aim was to consider the safety and feasibility of KT in PTSD cohorts. By synthesizing evidence from preclinical, clinical, observational, mechanistic, and prior review articles, this review intended to summarize evidence, generate new insights, and direct future research.

## 2 Methods

We conducted an integrative review. We intentionally chose this approach for its ability to synthesize evidence across various study methodologies (54, 55). We followed key principles from Whittemore and Knafl (55) in conducting this review. Given the scope and design of this integrative review, we also drew on certain recommendations from the Preferred Reporting Items for Systematic Reviews and Meta-Analyses (PRISMA; 56), without intentionally aiming to meet all criteria for systematic reviews.

### 2.1 Search strategy

Between January 9^th^ and January 14^th^, 2025, we conducted a comprehensive search of bibliographic databases and clinical trial registers. The bibliographic search included PubMed, Scopus, PsycArticles, PsycINFO, PSYNDEX, and MEDLINE. Search strings combined terms referring to KT (i.e., “ketogenic” OR “ketosis” OR “ketone*” OR “MCT” OR “medium-chain triglycerides” OR “Atkins diet” OR “LCHF” OR “KLCHF” OR “LC/KD” OR “low carbohydrate diet”) with terms referring to PTSD (i.e., “post-traumatic stress disorder” OR “PTSD” OR “posttraumatic stress disorder” OR “post traumatic stress disorder”).

Search fields were selected according to the technical options available in each database. Where possible, searches were restricted to titles and abstracts. PubMed permitted direct searching of both title and abstract fields. In Scopus, the closest corresponding search option comprised titles, abstracts, and keywords. For PsycINFO, PsycArticles, PSYNDEX, and Medline, the available title and abstract fields were searched through all feasible combinations. We did not apply filters for study design, publication type, or article type, at this stage in order to preserve search sensitivity and capture evidence across study designs, in line with the integrative review framework and the scope of the search question. We additionally searched clinical trial registries, including the International Clinical Trials Registry Platform (ICTRP), EU Clinical Trials Register, and ClinicalTrials.gov. Search-term combinations were adapted to the search capabilities of each register. These registry searches were undertaken to identify additional relevant clinical research.

### 2.2 Screening and eligibility

After record identification, duplicates were detected by the web-based tool “Rayyan” (57) and, upon confirmation, manually removed. Screening and eligibility assessments were conducted in an independent dual-review process. Records were screened for relevance based on their focus on both KT (e.g., ketogenic diet, ketone supplementation) and PTSD or closely related core symptom domains such as dissociation and chronically elevated high stress. This broader symptom-based approach was adopted to enhance screening sensitivity and reduce the likelihood of excluding articles that discussed the relationship between PTSD and KT in the full text but did not explicitly mention this connection in the abstract. Disagreements were resolved unanimously through discussion of the full-text articles, guided by whether the manuscript established a link between PTSD and KT.

Following screening, all preliminary included records underwent full-text eligibility assessment. Articles were eligible if they were (1) preclinical, clinical, mechanistic, observational, or review articles and (2) if they reported on biological mechanisms, clinical outcomes, participant experiences, neurobiological or other biological changes related to both KT and PTSD, or evidence relevant to evaluating the safety and feasibility of KT for PTSD. Eligibility disagreements were resolved through criterion-based discussion, with unanimous agreement reached for all final inclusion decisions.

### 2.3 Study quality assessment

After the eligibility assessment, we evaluated the quality of included studies. Given the range of study designs, a two-step blinded procedure was employed. First, the level of evidence was ranked based on well-established hierarchies of evidence (58, 59), which were adjusted to reflect the inclusion criteria of this review (see Table A in Supplemental Material). Second, each study’s individual methodological quality was rated, using the Critical Appraisal Tool (CAT; 60), selected for its validity, reliability, and suitability across diverse study designs (60). Results were rated on a three-point Likert scale (1 “low quality” – 2 “moderate quality” – 3 “high quality”). Qualitative discrepancies between raters were resolved by a third reviewer who made the final decision based on CAT criteria. Studies were not excluded based on quality ratings. Instead, study quality was considered and reflected in the synthesis process according to Whittemore and Knafl (55). Results from the study quality assessment are depicted in Table B in the **Supplementary Material**.

### 2.4 Data extraction and analysis

From eligible studies, we extracted data on KT and PTSD and organized findings on preliminary efficacy, feasibility, and safety into structured tables. We then summarized the data relating to each study aim and integrated the evidence while considering study quality. We next integrated the information on mechanistic pathways into a concise model depicting potential KT-induced alterations in PTSD pathophysiology. Lastly, we integrated the findings of the reviewed studies with the broader literature on PTSD to reflect on the research questions and to propose potential mechanistic downstream effects from KT-induced physiological alterations to the PTSD symptom-effect level.

### 2.5 Search update

**Figure 1:**
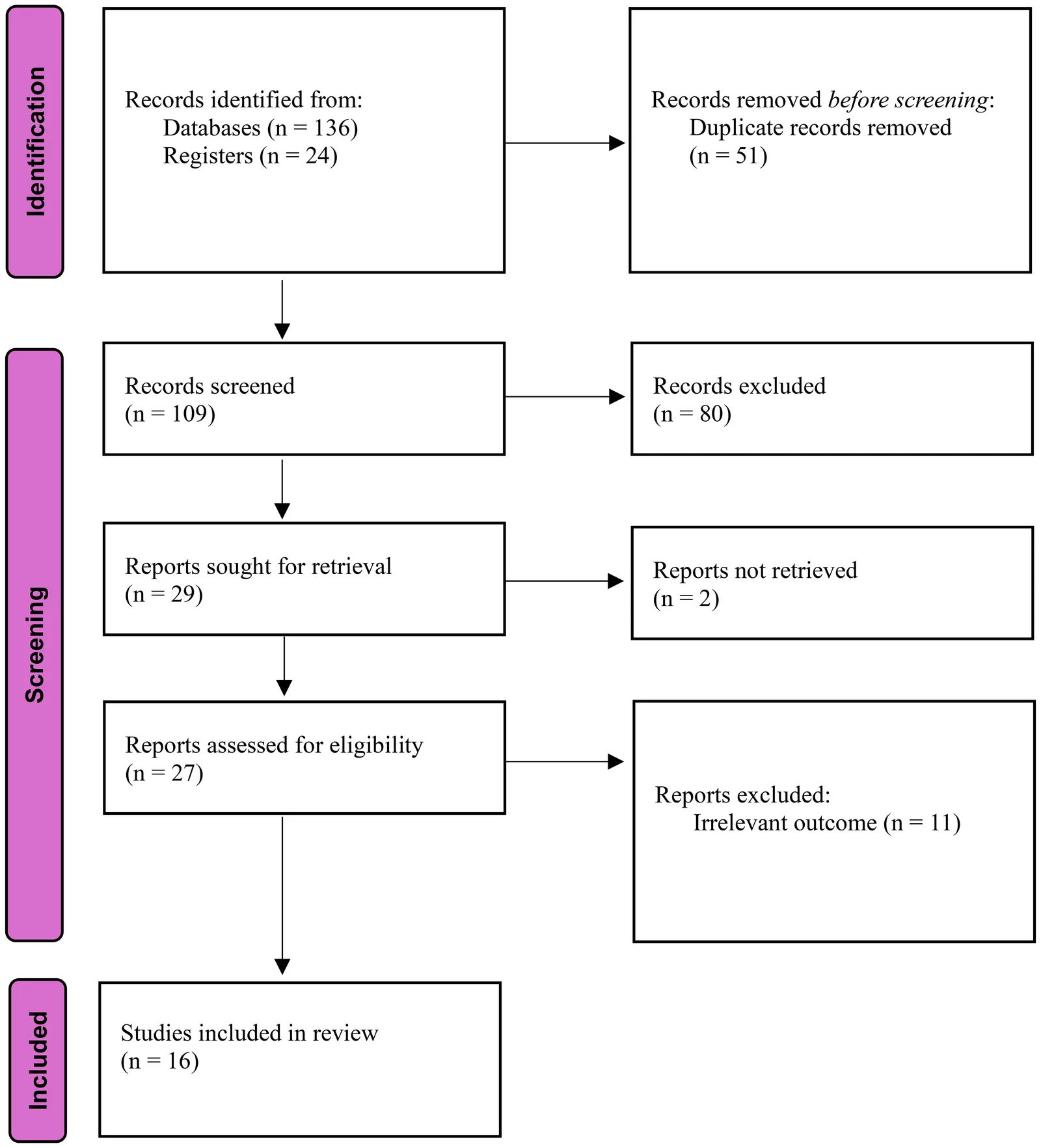
PRISMA flow diagram of the systematic search process. Source: Page MJ, et al. BMJ 2021;372:n71. doi: 10.1136/bmj.n71. This work is licensed under CC BY 4.0. To view a copy of this license, visit https://creativecommons.org/licenses/by/4.0/.

To ensure that this review reflects the most current scientific evidence, we conducted an updated search on May 4, 2026, during the peer-review process. The search update was performed in accordance with the original study protocol. We identified 32 potentially relevant records, of which 5 articles met the eligibility criteria and were included in the review. Separate flow diagrams for the initial search and the updated search are provided in the **Supplementary Material** (Figures A and B), whereas the flow diagram in the main manuscript (**Figure 1**) presents the combined results.

## 3 Results

The final sample comprised sixteen eligible publications (published 2020-2026): three clinical pilot trials, one cross-sectional quasi-case-control study, three preclinical studies, five human case reports, one mechanistic study, and three unsystematic mechanistic narrative reviews. Reviews and animal studies provided insights into how KT could mitigate PTSD pathophysiology. Preclinical studies assessed biological efficacy in rodent models, while case reports, observational studies, and pilot trials provided preliminary data on feasibility, safety, and efficacy signals in human patients with PTSD symptoms. Across interventional studies, applied interventions ranged from ketogenic diets to ketone injection, ketone salt, acetate supplementation, and combined approaches. While the methodological quality of all studies scored at least moderate, only two randomized-controlled pilot trials were categorized as providing high-level evidence by study design (see Supplemental Table B).

### 3.1 Safety analysis

We identified five studies reporting data that might be relevant to evaluate the safety of KT in PTSD populations: one twelve-week case report (61) and one 21-week case report (62) applying the ketogenic diet, a four-week uncontrolled pilot (63) combining the ketogenic diet with ketone salts, one cross-sectional observational study comparing Ukrainian youths with and without PTSD as well as their dietary patterns (64), and a six-week randomized placebo-controlled pilot trial (65) investigating ketone salt supplementation alone. Safety and feasibility results are summarized in **Table 1**.

**Table 1:** Key feasibility and safety outcomes from human studies (*N_Total_*= 724).

Reference	Design	Sample	Intervention	Key findings	Comments/conclusion
Anekwe et al. (61)	Case Report [direct clinical evidence]	1 patient	Ketogenic diet (12 weeks)	**Elevated LDL-C**^1^ **and liver enzymes:** AST^2^ increase from 21 U/L to 67 U/L ALT^3^ increase from 18 U/L to 119 U/L LDL-C^1^ increase from 156 mg/dL to 209 mg/dL HDL-C^4^ from 102 mg/dL to 98 mg/dL	Normalization after eight months, after prescribed atorvastatin 20 mg, vitamin D, omega 3 supplementation, additional dietary changes, and introduction of regular exercise. No PTSD-specific safety signals or feasibility obstacles.
Laurent (62)	Case Report [indirect human evidence]	1 patient	Ketogenic diet (21 weeks)	**Decreased carnitine** Low free carnitine after 3.5 weeks **Compliance rates** 89% for daily ketone measures 91% for daily glucose measures **Achievement of ketosis** Patient achieved nutritional ketosis	No PTSD-specific safety signals or feasibility obstacles.
Edwards et al. (63)	Pilot feasibility study [direct clinical evidence]	3 patients	Ketogenic diet and exogenous ketone supplementation (4 weeks)	**Feasibility** Identification rate: 40% Recruitment rate: 75% Completion rate: 67% Acceptance rate: 100% Compliance rates: 93-100% Achievement of ketosis: 100% **Adverse events/reactions** No serious adverse reactions or events Mostly mild adverse reactions on 70% of intervention days **Biochemical findings** No changes in minerals Transient increases in liver enzymes, c-reactive protein, and LDL-C^1^ observed in a subset of patients (first 2 weeks), while improving beyond baseline after 4 weeks in remaining patients.	The authors reported difficulties in identifying eligible patients (i.e., few being forwarded from contacted therapists), suggesting a wide variety of recruitment strategies should be applied in future clinical studies. No PTSD-specific safety signals or feasibility obstacles were reported.
Locklin et al. (65)	Pilot randomized clinical trial [direct clinical evidence]	21 patients	Exogenous ketone supplementation (6 weeks)	**No differences in:** Systolic blood pressure Diastolic blood pressure Mean arterial pressure Resting heart rate Complete blood count Comprehensive metabolic panel	No PTSD-specific safety signals or feasibility obstacles.
Halabitska et al. (64)	Cross-sectional observational study [indirect human evidence]	448 probable PTSD patients, 250 healthy controls	No intervention; self-reports of different dietary adherence including ketogenic diets.	**Dietary findings:** Individuals with probable PTSD were more likely to report adhering to several restrictive diets, including low-fat diets and ketogenic diets. **Biochemical findings** **(probable PTSD group)** Lower hemoglobin (*p* = .004), Lower red blood cells (*p* = .016) Lower hematocrit (*p* = .003) Lower mean corpuscular volume (*p* = .048) Lower mean corpuscular hemoglobin (*p* = .001) Higher white blood cell count (*p* = .045) Higher neutrophils (*p* = .006) Higher erythrocyte sedimentation rate (*p* = .017) Higher neutrophil-to-lymphocyte ratio (*p* <.001) Lower lymphocyte counts (*p* = .002)	This study was conducted as a cross-sectional observational study on Ukrainian youth with who scored above or below the PCL-54 cut-off score, indicating PTSD. The authors call the study a pilot case-control study. However, no intervention was applied, and group allocation has been applied *post-hoc* based on PCL-5 scores. The study may deliver limited indirect evidence regarding the feasibility and safety of the ketogenic diet for PTSD. All effect sizes were small.

^1^LDL-C, Low-Density Lipoprotein-Cholesterol; ^2^AST, Aspartate Aminotransferase; ^3^ALT, Alanine Aminotransferase; ^3^HDL-C, High-Density Lipoprotein-Cholesterol.

Only one uncontrolled pilot (*n* = 3) assessed adverse events and adverse reactions following the ketogenic diet (defined as a high-fat, moderate-protein, very-low-carbohydrate diet, aiming at capillary β-hydroxybutyrate ≥ 0.5; 63) and ketone salt supplementation in PTSD patients. No serious adverse reactions, unexpected adverse reactions, or serious adverse events were reported. Although patients reported several (mostly mild to moderate) adverse reactions during the intervention period (most frequently: headaches and fatigue), those could not be distinguished from the patients' pre-existing psychopathology (including co-occurring disorders/concerns) due to the lack of a control condition. The authors discussed adverse reactions as common symptoms during the adaptation period to the ketogenic diet (63). Importantly, no PTSD-specific safety signals were reported.

The reported data from biochemical assessments appear relevant in evaluating the safety of KT for PTSD. Such data were reported in four human studies that applied different forms of KT (61–63, 65). In a randomized controlled pilot trial (*n* = 21) investigating β-hydroxybutyrate salt supplementation, Locklin et al. (65) found no statistically significant group differences in systolic or diastolic blood pressure, mean arterial pressure, resting heart rate, complete blood count, and comprehensive metabolic panel, supporting its short-term (i.e., six weeks) safety in PTSD patients. With respect to the ketogenic diet, Anekwe et al. (61) report on a patient with obesity who self-administered the diet (described as consuming predominantly eggs, cheese, butter, oil, nuts, leafy green vegetables and almond/coconut milk; 61) for three months; around this period, aspartate aminotransferase (AST) rose from 21 U/L to 67 U/L, alanine aminotransferase (ALT) from 18 U/L to 119 U/L, and low-density lipoprotein-cholesterol (LDL-C) from 156 mg/dL to 209 mg/dL, high-density lipoprotein-cholesterol (HDL-C) decreased from 102 mg/dL to 98 mg/dL, and a vitamin D deficiency was also noted. These changes were interpreted by the authors as indicating non-alcoholic fatty liver disease. The parameters were measured five months before and three months after starting the ketogenic diet. They normalized eight months after halting the diet. Notably, regular exercise, atorvastatin (20 mg), vitamin D, and omega-3 supplementation were also introduced and not considered in the authors’ interpretation. In the article by Edwards et al. (63), individual biochemical trajectories varied. One patient showed declines in total cholesterol and LDL-C with stable liver enzymes and C-reactive protein (CRP). Another patient exhibited initial ALT elevation (62 to 115 U/L) that normalized by the fourth week. The third patient discontinued early after initial rises in LDL-C and CRP. Moreover, Laurent (62) reported free carnitine depletion in one patient at 3.5 weeks on the ketogenic diet (daily macronutrient ratio of: 154 g fat, 72 g protein, 30 g carbohydrates; 62).

For completeness, we also considered a Ukrainian cross-sectional study of young individuals (64). The study assessed probable PTSD status (based on psychometric threshold exceedance), blood-based biomarkers, and self-reported adherences to several dietary patterns including low-fat diets, high protein diets, ketogenic diets (here defined as carbohydrate intake < 10–15% of total calories, fat intake > 60% of calories), Mediterranean diets, vegetarian-vegan diets, and intermittent fasting (64).

Participants with probable PTSD (based on a PCL-5 score of 31-33) were more likely to show signs of physiological stress and immune activation/inflammation, particularly disruptions of the neutrophil-to-lymphocyte ratio (see **Table 1**). Such individuals were also more likely to report restrictive dietary patterns, including ketogenic diets. However, associations were only cross-sectional, and biological outcomes were not directly linked to the ketogenic diet. Moreover, given the broad dietary definitions, it remains uncertain whether the ketogenic diet subgroup achieved meaningful ketosis (64). Therefore, we posit these findings primarily reflect PTSD-related physiological alterations rather than ketogenic dietary effects.

Taken together, controlled data support the safety of exogenous β-hydroxybutyrate supplementation in PTSD patients over six weeks, while uncontrolled evidence suggests alterations in specific biochemical markers in some PTSD patients following different forms of ketogenic diets. The observed alterations might be insufficient to indicate safety signals – a point that will be elaborated in the discussion section. In summary, the unavailability of controlled data to date does not permit a robust safety assessment for PTSD. Accordingly, insights from related disciplines such as neurology may help to contextualize and guide interpretation.

### 3.2 Feasibility analysis

In addition to the reported data on the safety of KT for PTSD, two records explicitly reported feasibility outcomes for PTSD: a four-week pilot combining the ketogenic diet with exogenous ketone supplementation (63) and a single-case study applying the ketogenic diet (62). In the pilot trial, the authors reported their intent to identify ten eligible patients, while achieving only four (40%), of whom three were recruited (75% of those identified), and two completed the four-week protocol (67% completion of those initiating the trial; 63). The authors attributed the difficulties in participant identification to the limited scope of their recruitment strategies rather than to PTSD-specific barriers. The dropout of one participant after two weeks was attributed to the combined burden of meal preparation, measurements, and registration, which also appears not to be specific to PTSD. Across both studies, all participants achieved nutritional ketosis (which they defined as serum β-hydroxybutyrate > 0.5 mmol/L; 62, 63). In the pilot, ketosis was maintained on 87% of intervention days, while all participants accepted the treatment on 100% of the intervention days (63). Dietary logs were completed 93-100% of the time in the pilot, and ketone/glucose measurements were submitted on 89% of ocassions in the pilot and in 91% of ocassions in the case report (62, 63). Participants’ perceptions of burden varied from “not demanding” to “quite to very demanding,” and the patient from the case report described adherence to the ketogenic diet as sustainable to the extent required for symptom management (62, 63).

Additional indirect evidence on feasibility might be concluded from case- and observational studies. Halabitska et al. (64) demonstrated that among 448 individuals with probable PTSD, 66 individuals reported to have adapted a ketogenic-diet-like dietary pattern, suggesting that such an approach may be implementable in this population (64). Moreover, three additional case studies indicate that individuals with a PTSD diagnosis or PTSD symptoms (*N_Total_* = 4) were able to follow a ketogenic diet over several weeks, while also adhering to glucose and ketone monitoring protocols and completing symptom questionnaires (66–68).

In summary, the few existing data seem to overall support the feasibility of KT in PTSD patients, while some aspects (i.e., recruitment, participant burden) likely need enhanced attention when planning clinical KT research. To date, there were no indications of PTSD-specific constraints reported.

### 3.3 Mechanistic analysis

To analyze the mechanistic data on KT for PTSD, we synthesized findings from three narrative reviews (69–71), four rodent studies (72–75), and one human study (63). Together, these publications suggest that KT may address PTSD-specific pathophysiological factors related to metabolic pathways, ketone-signaling properties, and neurotransmitter regulation.

#### 3.3.1 Metabolic considerations

Three narrative reviews (69–71) and one mechanistic study (72) suggest PTSD-associated disruptions in brain energy metabolism and mitochondrial function, while indicating KT may restore bioenergetic homeostasis.

Turkson et al. (71) outlined how chronic HPA axis and sympathetic overactivation may impose an allostatic load that can overwhelm neuronal mitochondria and compromise ATP production. Relatedly, Preston et al. (72) demonstrated that rodents developing PTSD-like symptoms displayed reduced markers of mitochondrial bioenergetics alongside altered phospholipid metabolism, suggesting impaired ATP production. Furthermore, Gary et al. (70) reported that chronic increases in serum glucose and insulin through stress could, in the long term, dysregulate glucose metabolism. In this regard, Turkson et al. (71) highlighted that PTSD has been associated with disruption of glucose transporters (GLUT)^1^ involved in transporting glucose from serum to the brain (through the blood-brain barrier). This builds on early evidence from Garvey et al. (79), who first identified glucose transporter (GLUT) malfunctions in rat and adipocyte cell cultures.

El Karkafi et al. (69) discussed that the ketogenic diet may reverse mitochondrial dysfunction by shifting energy production toward ketone-driven ATP synthesis. Similarly, Turkson et al. (71) discussed ketone bodies as an alternative fuel source for the brain that could account for stress-related deficiencies. In this regard, Preston et al. (72) demonstrated rodents subjected to a PTSD-induction paradigm displayed depleted cerebellar adenosine monophosphate and adenosine diphosphate pools alongside elevated plasma β-hydroxybutyrate and decreased branched-chain amino acids, consistent with an attempt to solve an energy crisis and stabilize trauma-induced energy disruptions (72). Furthermore, Gary et al. (70) summarized findings on significant elevations of β-hydroxybutyrate in serum and the prefrontal cortex following acute stress, suggesting an endogenous shift toward ketone utilization as a compensatory response to energy deficits.

Having demonstrated that ketone bodies can pass the blood-brain barrier to be mobilized when needed, Preston et al. (2021, pp. 8-9) directly suggested “that ketogenic diet or exogenous ketone body supplementation may offer therapeutic potential for patients with PTSD.” Taken together, these studies suggest a biological rationale for the therapeutic potential of KT on PTSD pathophysiology. Specifically, KT may influence metabolic pathways to address PTSD-associated brain energy deficits and mitochondrial dysfunction.

#### 3.3.2 Ketone signaling properties

In addition to metabolic effects, ketone signaling properties may address PTSD-specific pathophysiological alterations, such as inflammation (69–71), oxidative stress (69, 70, 72), neuronal survival and growth, brain-derived neurotropic factor (BDNF) impairments (75), and neuronal damage (69–71). El Karkafi et al. (69) described ketogenic diet-induced anti-inflammatory effects due to the inhibition of the nuclear factor kappa-light-chain-enhancer of activated B cells (NF-κB). They further mentioned the activation of the peroxisome proliferator-activated receptor gamma (PPARγ), which would also reduce neuroinflammation. Another review (70) highlighted the ability of the ketone body β-hydroxybutyrate to inhibit the NOD-like receptor family pyrin domain-containing protein 3 (NLRP3) inflammasome (80), thereby downregulating the release of pro-inflammatory cytokines. El Karkafi et al. (69) summarized evidence that ketogenic diets reduce levels of pro-inflammatory cytokines (e.g., interleukin-1 beta [IL-1β], interleukin-6 [IL-6], and tumor necrosis factor alpha [TNF-α]).

Two preclinical rodent studies and one human pilot provide empirical support for these mechanisms in the context of KT for PTSD (63, 73, 74). In a single prolonged stress (SPS) model (*n =* 18), Yamanashi et al. (74) reported that β-hydroxybutyrate injection resulted in significant reduction of trauma-induced serum TNF-α elevation (Mann–Whitney U-test; W = 69, *p* = .039), though without statistically significant evidence for a change in IL-1β concentration (Mann–Whitney U-test; W = 49.5, *p* = .33). Changes were accompanied by attenuated anxiety-like behaviors.

Similarly, Tanelian et al. (73) reported that sodium-acetate administration (raising endogenous β-hydroxybutyrate fourfold in the SPS condition) in rodents (*n* = 14) downregulated hippocampal IL-1β gene expression (*p* = .0004, 73) and decreased Iba1+ cells (*p* = .0439, 73) in the ventral hippocampus. Effects were accompanied by improvements in stress-related behavior. In contrast, Yoshioka et al. (75) found that MCT oil supplementation elevated β-hydroxybutyrate levels in rodents but did not significantly affect cytokine levels, whereas continuous supplementation did reduce anxiety-like behaviors (75).

Edwards et al. (63) examined C-reactive protein (CRP) in PTSD patients undergoing a ketogenic diet with exogenous β-hydroxybutyrate supplementation. While transient increases during the first two weeks were indicated in two patients, those completing the intervention experienced not only normalization (i.e., < 5) but improvement beyond baseline (63).

Other potential ketone signaling effects include activation of the hydroxycarboxylic acid receptor 2 (HCA2) and the nuclear factor erythroid 2-related factor 2 (Nrf2) pathway, both potentially attenuating PTSD-associated oxidative stress and cellular damage (69–71). El Karkafi et al. (69) discussed the binding of β-hydroxybutyrate to the HCA2 receptor to induce additional neuronal protective functions by modulating a neuroprotective phenotype in macrophages. Neuroprotective properties of the ketogenic diet, described by Turkson et al. (71) and Gary et al. (70), suggest that activation of the Nrf2 pathway may enhance cellular defenses against reactive oxygen species, thereby reducing pro-apoptotic signaling.

Two reviews and one rodent study provide supplemental information on the role of oxidative stress in KT and PTSD (69, 70, 72). El Karkafi et al. (69) discussed stress-associated increases in reactive oxygen species, which, according to the authors, negatively impact the functioning of the amygdala, the prefrontal cortex (PFC), and the insula (69).^2^ The authors propose the potential of KT to reduce the production of reactive oxygen species. In support of these assumptions, Preston et al. (72) found that stress-susceptible rodents (identified by SPS – a modified version of the Stress-Enhanced Fear Learning [SEFL] paradigm exposure consisting of inescapable foot shock exposure) exhibited significantly lower cerebellar taurine – a mitochondrial antioxidant buffer – compared to stress-resilient animals, suggesting elevated oxidative stress in PTSD-vulnerable animals.

Another potential β-hydroxybutyrate signaling effect may involve BDNF regulation. Yoshioka et al. (75) reported significantly reduced BDNF response in rodents under acute stress following continuous MCT oil supplementation that implicated plasma elevation of β-hydroxybutyrate levels.

Overall, the available data suggest that KT-related signaling pathways may influence interconnected biological processes implicated in PTSD, including inflammation, oxidative stress neuronal damage, and BDNF-related mechanisms.

#### 3.3.3 Neurotransmitter regulatory effects

Another pathway, described in two reviews and one rodent study, involves regulation of specific neurotransmitters (i.e., GABA and glutamate) that may improve PTSD (69, 70, 72). Gary et al. (70) described recurring high stress triggering increased glucocorticoid release, which is associated with glutamate increases in limbic and cortical regions. These would result in increased neuronal excitability. As glutamate acts on various receptors, including N-methyl-D-aspartate and α-amino-3-hydroxy-5-methyl-4-isoxazolepropionic acid, the authors propose that elevated glutamate may contribute to disturbances in learning and memory processes (70).^3^ Moreover, the authors posit that increased glutamate in conjunction with the activation of the HPA axis and cortisol release could amplify stress and emotional responses. Both El Karkafi et al. (69) and Gary et al. (70) mentioned KT’s potential to increase GABA in relation to glutamate. Relevant in the context of PTSD, El Karkafi et al. (69) also reported that increased GABA may result in calming effects, improved sleep quality, and reduced anxiety. In alignment with these effects, Preston et al. (72) noted that KT may reduce neuronal hyper-excitability through modulation of the GABA-glutamate balance. Considering that GABA may serve as a “physiological antagonist” to glutamate^4^, KT may reduce PTSD symptomatology via GABA-induced reductions in neuronal excitability, which may have downstream psychological effects.

### 3.4 Preliminary efficacy analysis

Preliminary efficacy data of KT for PTSD stems from six human (62, 63, 66, 86) and three preclinical animal studies (73–75). Only a few studies investigating the therapeutic efficacy of KT for PTSD seem to have been conducted to date. However, the six identified human records report psychometric assessments of PTSD symptoms before and after the introduction of KT, while preclinical records consistently report KT-induced behavioral changes in animal models. A summary of the preliminary efficacy evidence from human and animal studies can be found in **Table 2**.

**Table 2:** Preliminary efficacy data from preclinical (n_Rodents_ = 93), and human intervention studies (n_Humans_ = 25).

Reference	Design	Cohort/model	Intervention	Psychometric and behavioral outcomes	Comments/conclusions
Tanelian et al. (73)	Preclinical Study [preclinical/ mechanistic evidence]	*N* = 48 rodents *n*_sps_^1^ = 28	Sodium acetate supplementation (*n* = 14)	**Behavioral tests** Decreased anxiety-like and avoidance behaviors and improved social behavior in intervention group with SPS^1^ compared to control group with SPS^1^: Decreased anxiety and avoidance behavior was statistically significant in Open Field and Elevated Plus Maze tests; improved social behavior was observed in the Social Interaction test.	Acetate supplementation led to 4-fold increased serum ketone concentrations in stressed rodents compared to unstressed rodents. Intervention had no impact on unstressed controls. Alteration in stress response was speculated to have been impacted by conversion of acetate to β-hydroxybutyrate.
Yamanashi et al. (74)	Preclinical Study [preclinical/ mechanistic evidence]	*N* = 36 rodents *n_SPS_*^1^ = 18	Exogenous ketone (β-hydroxybutyrate) injection (*n* = 9)	**Behavioral tests** Decreased post-traumatic anxiety behavior observed in the intervention group with SPS^1^, indicated by Open Field (OFT) and Elevated Plus Maze (EPM) tests.	Alterations through β-hydroxybutyrate were trauma-specific. Supplementation of β-hydroxybutyrate alone did not yield anxiolytic effects.
Yoshioka et al. (75)	Preclinical Study [preclinical/ mechanistic evidence]	*N* = 16 rodents *_nMCT2_* = 5 *n_LCT_^3^* = 4	MCT^2^ oil supplementation (*n* = 5)	**Behavioral tests** Decreased post-traumatic (SPS^1^) anxiety-like behavior observed in the MCT group (Elevated Plus Maze (EPM) and Open Field Tests (OFT). Serum brain-derived neurotropic factor (BDNF) was significantly lower under acute stress in the MCT group.	MCT-associated anxiolytic effects were specifically trauma-related. Under acute stress in subsequent experiments, serum BDNF levels were significantly lower in the MCT condition.
Laurent (62)	Case Report [indirect human evidence]	1 patient with bipolar disorder and PTSD symptoms	Ketogenic diet (21 weeks)	**Psychometrics** PCL-5^2^: Total score (38→8) D criterion (16→3) E criterion (14→2)	The study is on a patient with bipolar disorder II diagnosis; it has been included due to PTSD symptom reports pre and post ketogenic dietary intervention, and particularly because PCL-5^2^ scores were consistent with PTSD.
Laurent (67)	Case Report [direct clinical evidence]	1 patient with PTSD	Ketogenic diet (25 weeks)	**Psychometrics** PCL-5^2^: Total score (32→2) B criterion (5→0) C criterion (6→1) D criterion (15→1) E criterion (6→0)	The structured dietary intervention seems to have lasted for 25 weeks, although a final assessment in week 27 is mentioned.
Bellamy & Laurent (66)	Case Report [direct clinical evidence]	1 highly comorbid patient with PTSD	Ketogenic metabolic therapy^4^ (12 weeks)	**Psychometrics** PCL-5^2^: Total score (80→0)	Full remission of PTSD and psychiatric comorbidity after the 12-week intervention reported. The patient reports optimal symptom management at β-hydroxybutyrate levels between 3.0 and 5.0 mmol/L.
Laurent et al. (68)	Case series [indirect human evidence]	2 patients with schizoaffective disorder and PTSD symptoms.	Ketogenic diet (24 weeks)	**Psychometrics** PCL-5^2^: Patient 1 (38→8) Patient 2 (8→3)	The study is on two patients with schizoaffective disorder. It has been included due to PTSD symptom assessment and specifically because PCL-5^2^ scores of patient 1 were consistent with PTSD. Final scores have been assessed at week 24 in Patient 1 and after continuation at week 27 in Patient 2.
Edwards et al. (63)	Pilot feasibility study [direct clinical evidence]	3 patients with PTSD, 2 study completes	Ketogenic diet and exogenous ketone salt (β-hydroxybutyrate) supplementation (4 weeks)	**Psychometrics** PCL-5^2^: Patient 1 (70→50) Patient 2 (50→40) Patient 3 discontinued.	The average reduction in PCL-5^2^ scores was 25%, but no control condition existed.
Youssef et al. (86)	Pilot RCT [direct clinical evidence]	21 patients with PTSD	Exogenous ketone salt (β-hydroxybutyrate) supplementation (6 weeks)	**Psychometrics** PCL-5^2^ median: Intervention group (58.5→54, *p* = .0003, *n* = 11) Control group (34→34, *p* = .4418, *n* = 9) Median group differences: Pretest (*p* = 1.0) Posttest (*p* = .60)	Only median differences were tested, not mean scores; while medians did not change, means also numerically decreased in the control group.

^1^SPS, single prolonged stress; ^2^PCL-5, PTSD Checklist for DSM-5. ^2^MCT, medium-chain triglycerides; ^3^LCT, long-chain triglycerides ^4^The keto-metabolic therapy intervention in this case included the ketogenic diet, regular exercise, and intermittent fasting.

#### 3.4.1 Preclinical evidence

In preclinical studies applying an SPS model for PTSD, supplementation and injection associated with increased serum β-hydroxybutyrate resulted in statistically significant PTSD-like symptom improvement (73–75). Yamanashi et al. (74) directly injected β-hydroxybutyrate, which resulted in reduced post-traumatic anxiety-like behavior (i.e., more time spent in open arms of an elevated plus maze). The researchers proposed ketone supplementation as a potential novel approach for treating PTSD. Yoshioka et al. (75) supplemented MCT oil, a precursor of β-hydroxybutyrate, which can be easily transformed to, and led to increases in β-hydroxybutyrate levels and was associated with less anxiety-like behavior (in plus maze test), whereas changes in open-field tests were not statistically significant (75).

Tanelian et al. (73) supplemented sodium-acetate, the salt form of short-chain fatty acid, in a controlled setting. Sodium acetate is rapidly internalized by cells and directly converted into acetyl-CoA by the enzyme acetyl-CoA synthetase. The researchers initially based their hypotheses on microbiota-related effects of acetate supplementation. However, they recognized that stressed rats in the acetate condition showed a four-fold increase in serum β-hydroxybutyrate concentration compared to control conditions. These rats showed reduced anxiety-like behaviors (i.e., more frequent entries, more time spent in the open arms, and less frequent head dips during the elevated plus maze test), improved social behaviors (i.e., shorter latency to first approach, more time spent, and more interactions during the social interaction test), accompanied by decreased biological stress responses to the SPS (i.e., lower corticosterone and epinephrine levels). The researchers speculated that in stressed animals, acetate was converted to β-hydroxybutyrate, which through improved energy supply inhibited the response of the sympathetic nervous system, effectively reducing anxiety. They discussed the ketone body β-hydroxybutyrate as a potential mediator between acetate supplementation and PTSD-specific symptom improvement (73).

In summary, preclinical evidence suggests potential efficacy of interventions that raise serum β-hydroxybutyrate in attenuating post-traumatic behavioral impairments, biological stress responses, and anxiety symptoms. Preclinical studies also deliver evidence for the ketone signaling and brain energy pathways described previously.

#### 3.4.2 Evidence from human studies

Psychometrically assessed PTSD symptom scores from six human studies deliver only preliminary efficacy signals regarding KT for PTSD. In a small pilot double-blind randomized placebo-controlled pilot trial (86), exogenous ketone salts were supplemented for six weeks (*n_IG_*= 11) and examined in comparison to a placebo control group (*n_CG_*= 9). Post-assessment PTSD Checklist for DSM-5 (PCL-5) score medians were significantly lower in the intervention group compared to pre-assessment scores (*p* = .0003), while such an effect was not found in the control group (*p* = .4418; 86). Importantly, median group differences between the intervention and the control condition were not statistically significant and therefore did not establish clinical efficacy. In a pilot feasibility study with three patients following a four-week ketogenic diet with additional ketone supplementation, two patients completed pre- and post-intervention PCL-5 assessments (63). Average PCL-5 scores numerically decreased by 15 points. Furthermore, qualitative examination and psychometric measures indicated overall improvements in life quality among patients undergoing KT.

Bellamy and Laurent (66) reported on a patient with PTSD and multiple psychiatric comorbidities. After 12 weeks of a ketogenic metabolic intervention, incorporating ketogenic diet, exercise, and intermittent fasting to increase ketone levels progressively, the patient showed complete PTSD symptom remission, with PCL-5 scores decreasing from 80 (maximum) at baseline to 0 (minimum) at the final assessment. Laurent (67) reported another PTSD case in which PCL-5 symptom scores decreased from 32 to 2 after 27 weeks of following a ketogenic dietary intervention.

Additional indirect clinical evidence comes from two publications reporting PTSD symptom trajectories in patients with primary diagnoses other than PTSD. Laurent (62) reported a patient with bipolar II disorder following 21-week ketogenic diet, and Laurent et al. (68) reported two patients with schizoaffective disorder following a 24-week ketogenic dietary intervention. Two of these three patients initially scored above the PCL-5 clinical threshold for PTSD (62, 68, 87). In all three cases, PCL-5 scores decreased and reached subclinical levels by the end of the interventions (62, 68).

In summary, observational evidence and numerical decreases in psychometric PTSD scores from six human studies (although with very small sample sizes) together with preclinical evidence (suggesting behavioral alterations) seem to suggest preliminary efficacy signals of KT for PTSD, while definitive efficacy in large human trials remains to be tested.

## 4 Discussion

To our knowledge, this integrative review is the first to focus on the intersection between KT and PTSD. We conducted this review to explore the potential of KT as a treatment for PTSD, while considering evidence regarding KT’s PTSD-specific safety and feasibility. Although the evidence base is small and dominated by reviews, animal studies and uncontrolled human pilot trials, we were able to preliminarily evaluate relevant safety and feasibility aspects. We also identified preliminary efficacy signals from human and animal studies, and several plausible pathways through which KT may attenuate PTSD-associated pathophysiology. We posit this review to be particularly relevant as its findings can serve to inform future clinical research studies.

### 4.1 Safety considerations

Our synthesis of the available safety data suggests that, while short-term exogenous ketone supplementation appears safe in patients with PTSD (65), ketogenic diet may involve transient metabolic alterations. Specifically, ketogenic diets (implemented solely or combined with ketone salts) have been associated with fluctuations in liver enzymes, lipid profiles, carnitine levels, and inflammatory markers (61–63). The transition to ketosis entails a profound metabolic shift (88, 89); thus, alterations in metabolic biomarkers are unsurprising. Indeed, the metabolic alterations described in Anekwe et al. (61), Edwards et al. (63), and Laurent (62) correspond to well-characterized biomarker fluctuations during keto-adaptation that usually normalize with sustained adherence to the ketogenic diet (88–93). The mostly mild to moderate adverse reactions (e.g., fatigue and headache) described by Edwards et al. (63) and the declines in free carnitine levels described by Laurent (62) appears consistent with well-documented, transient changes during initial ketosis-adaptation (89, 90).

Notwithstanding the interpretations in the case report from Anekwe et al. (61), we view the attribution of elevation in LDL-C and AST/ALT to ketogenic diet-induced non-alcoholic fatty liver disease with considerable reservation. While the transient nature of these symptoms appears likely after prolonged ketosis (89, 92, 93), the authors’ interpretation is significantly undermined by several recent reviews proposing the ketogenic diet as a strategy to treat non-alcoholic fatty liver disease (94–96). Moreover, the timing of the initial laboratory evaluation completed five months prior to the study raises concern for causation. Furthermore, the authors did not account for reported co-occurring disorders/concerns, concurrent medications, medication changes, vitamin deficiencies, and lifestyle changes in their attribution solely to the ketogenic diet (61). Interestingly, in this context, Edwards et al. (63) subsequently observed that prolonged adherence to the intervention yielded improvements in LDL-C, CRP, and liver enzymes beyond baseline, despite transient increase during the first two weeks. Emerging evidence also suggests that ketogenic-diet-induced elevation in LDL-C does not carry the same atherogenic risks as comparable increases following standard diets (97–99). For instance, LDL-C elevation through ketogenic diet remained unassociated with coronary plaque burden, atherosclerosis, and heart disease (97–99).

In summary, methodologically high-quality controlled data (although preliminary and with small sample size) support short-term physiological safety of β-hydroxybutyrate supplementation, while biomarker alterations described in lower quality evidence (especially uncontrolled studies) may be attributed to metabolic changes during keto-adaptation. Additionally, no PTSD-specific safety signals were reported. Since reported symptoms appear not to be specific to PTSD and – due to missing control conditions - they may not be causally attributed to the ketogenic diet, we suggest cautiously reverting back to epilepsy research on the safety of the ketogenic diet, which suggests safety for most patients (100, 101). Although transient symptoms may occur, existing data indicate alterations during the adaptation to the ketogenic diet not to be harmful (89, 92, 95–101).

### 4.2 Feasibility considerations

The few studies that recorded feasibility outcomes on KT for PTSD suggest the intervention is overall implementable (62, 63). Challenges reported were difficulties enrolling sufficient participants and a single drop-out from a small study (63). These challenges were attributed to narrow recruitment strategies and a demanding study protocol, potentially reflecting the specific methodology of the study rather than infeasibility of KT for PTSD (63). While both challenges should inform future research, they do not appear to be unique to PTSD. In addition, several studies in individuals with clinically diagnosed PTSD or substantially elevated psychometric PTSD symptom scores who adopted ketogenic diets or related dietary patterns (64, 66, 67) may provide indirect, preliminary evidence that KT may be feasible in PTSD populations. Overall, these preliminary findings suggest that ketogenic dietary implementation may be feasible in PTSD populations, whereas more dedicated feasibility studies appear necessary to confirm this possibility.

### 4.3 Preliminary efficacy

While high-level clinical evidence on KT in large, controlled trials of human PTSD patients was absent, the existing preclinical, observational, and mechanistic evidence might be suggestive of potential benefits of KT for PTSD. Preclinical data from PTSD animal models specifically indicate early-stage efficacy signals of approaches that lead to increases in serum ketone (i.e., β-hydroxybutyrate) availability. Particularly, supplementation with MCT oil, acetate, and injection of β-hydroxybutyrate have led to reductions in post-traumatic stress and anxiety-like behavior in rodents (73–75). In human studies, numerical reductions in PTSD symptom scores following different forms (e.g., ketogenic diets and ketone supplementation) and durations (i.e., four, six, twelve, twenty-one, twenty-five weeks) of KT have been reported, albeit not in large randomized controlled trials (62, 63, 66–68, 86). Thereby, it is notable that one case report (62) and one case series (68) report only indirect PTSD evidence since these patients were not reported to have been formally diagnosed with PTSD, although PTSD symptom scores were assessed.

Reductions in trauma- and anxiety-related symptomatology seem to align between different designs of human and animal studies (62, 63, 66–68, 73–75, 86) and with previous research on the ketogenic diet (reviewed in 102).

Notably, ketone-associated anti-anxiety effects in PTSD animal models were not stand-alone effects, but appeared specifically trauma-related (73–75). These findings may point to ketones bridging stress-related brain energy deficiencies leading to increased ATP availability in deficient brain regions (70) as was also discussed in reviews by Preston et al. (72) and Turkson et al. (71). In addition, observed anxiolytic effects may relate to KT-induced reductions in oxidative stress, neuroinflammation, and neuronal excitability, as well as related increases in GABA, as described by El Karkafi et al. (69) Turkson et al. (71), and Gary et al. (70).

Although preliminary KT efficacy data may appear suggestive of potential benefits for PTSD, evidence from clinical studies in humans remains scarce. Existing studies are limited by very small sample sizes and heterogenous KT approaches, and most notably, no efficacy for PTSD in humans was demonstrated against a control group. Another open question remains whether supplementation can achieve effects comparable to ketogenic diets. While supplementation may rapidly elevate circulating ketones, dietary interventions may exert more sustained effects and influence glucose metabolism (103) or the gut microbiome (104) more profoundly. But a great heterogeneity between dietary approaches, even in the current sample, complicates comparability. To establish efficacy, future clinical research is needed to systematically test various forms of ketogenic therapy in large randomized controlled clinical trials.

### 4.4 Mechanistic model

**Figure 2:**
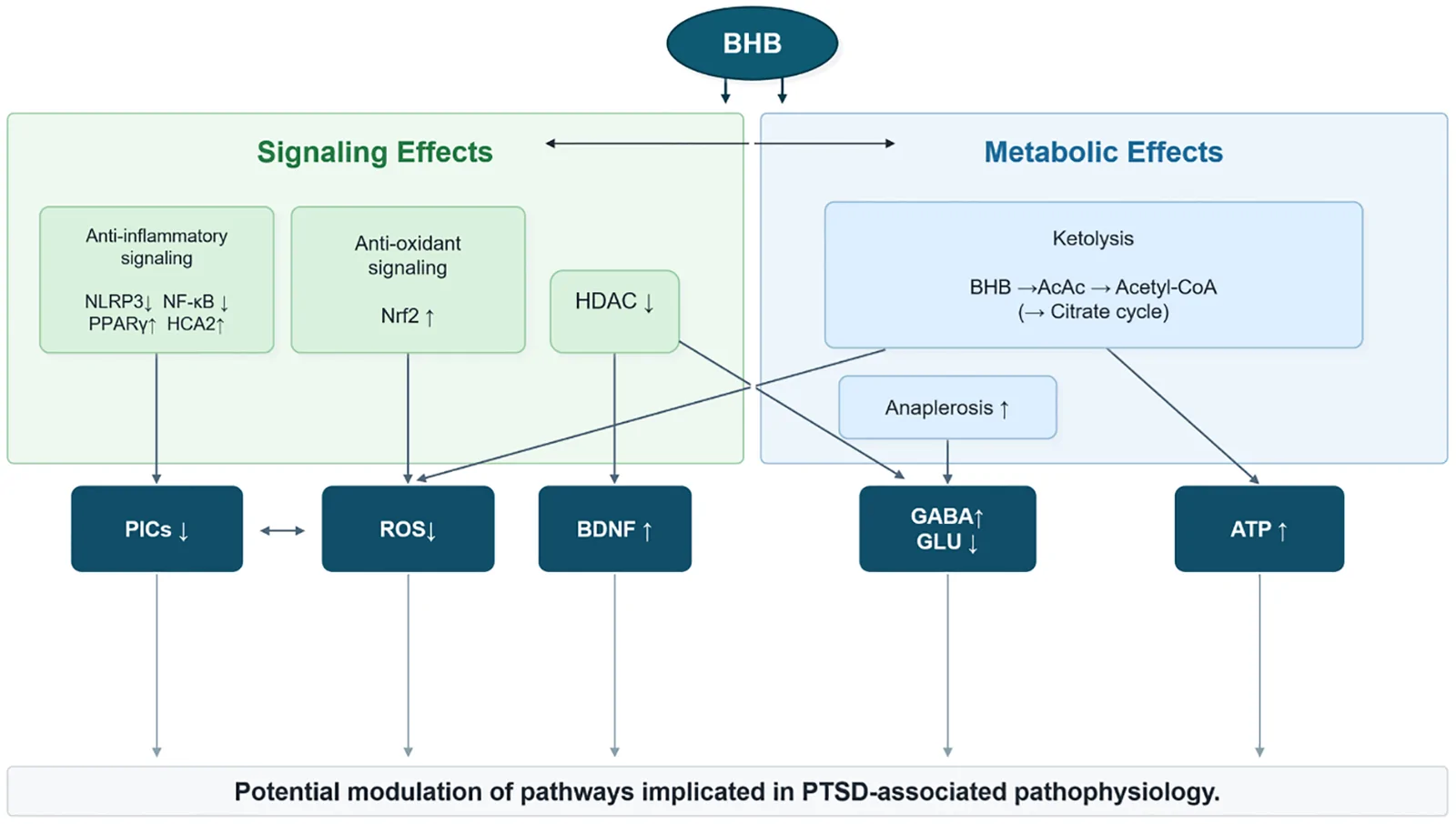
Conceptual model: potential physiological effects of ketogenic therapy on PTSD pathophysiology. BHB, β-hydroxybutyrate; NLRP3, NOD-like receptor family pyrin domain-containing 3; NF-κB, nuclear factor kappa-light-chain-enhancer of activated B cells; PPARγ, peroxisome proliferator-activated receptor; HCA2, hydroxycarboxylic acid receptor 2; Nrf2, Nuclear factor erythroid 2–related factor 2; HDAC, histone deacetylase; AcAc, acetoacetate; Acetyl-CoA, acetyl coenzyme A; PIC, pro-inflammatory cytokines; ROS, reactive oxygen species; BDNF, brain-derived neurotropic factor; GABA, gamma-aminobutyric acid; GLU, Glutamate; ATP, adenosine triphosphate.

**Figure 3:**
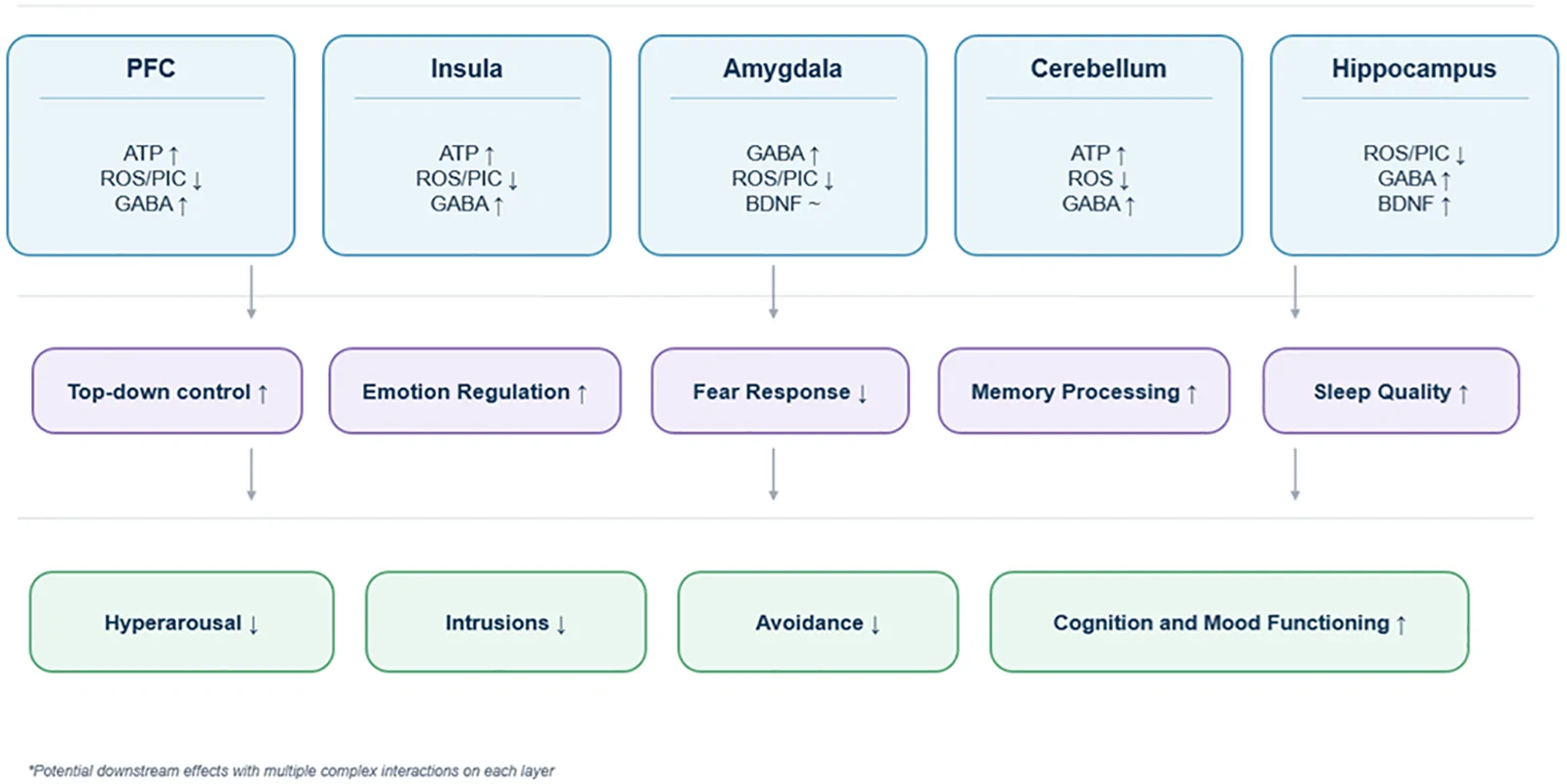
Conceptual model: potential downstream effects of ketogenic therapy – induced physiological alterations to PTSD symptomatology. PFC, prefrontal cortex; ATP, adenosine triphosphate; ROS, reactive oxygen species; PIC, pro-inflammatory cytokine; GABA, gamma-aminobutyric acid; BDNF, brain-derived neurotropic factor. Downstream effects constitute multiple complex interaction on and between each layer. This depiction is not intended to be exhaustive.

A substantial proportion of the information derived from included publications relates to mechanistic considerations and mechanistic evidence on the effects of KT on PTSD-related pathophysiology. By integrating these findings with the broader scientific literature on KT and PTSD, we have composed a mechanistic model (**Figure 2**) that may help understand how KT might impact PTSD pathophysiology. Based on this model and the broader literature, we also posit a cascade of potential downstream effects from KT-induced physiological alterations in PTSD-impacted brain regions that could influence PTSD-specific psychopathology (**Figure 3**). Importantly, this modeling approach should be understood as conceptual and hypothesis-generating rather than as conclusive depiction of causal pathways.

#### 4.4.1 Potential effects on PTSD pathophysiology

The mechanistic evidence from the reviewed studies suggests KT’s impact on PTSD pathophysiology may originate from the increased availability of the ketone body β-hydroxybutyrate. As outlined in the results section, β-hydroxybutyrate possesses specific signaling properties (Pathway 1) and fundamentally alters the metabolism of both body and brain (Pathway 2).

The signaling properties of β-hydroxybutyrate include activation of the PPARγ, HCA2 receptor, and Nrf2 pathway, as well as attenuation of the NLRP3 inflammasome and NF-κB pathway (69–71). While the effects on the PPARγ, HCA2, NF-κB, and NLRP3 include reducing pro-inflammatory cytokines (105–109), Nrf2 activation effectively regulates reactive oxygen species (110). Hereby, it is important to note that increases in reactive oxygen species and pro-inflammatory cytokines do not occur independently from each other, but interact reciprocally (i.e., more reactive oxygen species lead to more pro-inflammatory cytokines and vice-versa; 111, 112).

Furthermore, ketone-based interventions may rescue trauma-induced neurotrophic factor deficits. This mechanism is of particular relevance to PTSD as BDNF disruptions appear to be implicated in the disorder’s pathophysiology (113, 114). The available evidence suggests a complex pattern of BDNF dysregulation, whereby PTSD may be associated with lower baseline serum/plasma BDNF concentrations but elevated BDNF response to acute stress (75, 113, 115). By elevating β-hydroxybutyrate, ketone-based interventions may engage HDAC-related signaling cascades that might help restore BDNF regulation (116, 117) and could support the neuroplastic processes required for adaptive recovery following trauma (118).

Consistent with this hypothesis, elevating circulating β-hydroxybutyrate (BHB) via MCT administration has been shown to reduce anxiety-like phenotypes in the SPS model of PTSD, a behavioral improvement that coincides with the modulation of serum brain-derived neurotrophic factor (BDNF) responses (75). This suggests that the neuroprotective profile of the ketogenic diet may extend beyond metabolic rescue, potentially supporting the neuroplasticity required for functional recovery in PTSD (118). This interpretation converges with human studies suggesting that KT may positively affect BDNF regulation (119–121).

Metabolically, β-hydroxybutyrate promotes ketolysis, which through subsequent transformation via acetoacetate (AcAc) and Acetyl-Coenzyme A *(*Acetyl-CoA) activates the citrate cycle (122). This not only leads to increased ATP availability but also produces fewer reactive oxygen species in comparison to glucose-based ATP generation (123, 124).

Importantly, signaling and metabolic effects, although distinguishable, are not expected to occur independently from each other. For example, signaling properties of β-hydroxybutyrate (e.g., activation of HCA2; 125) are dependent on β-hydroxybutyrate concentrations which are themselves determined by metabolic processes (125, 126). Furthermore, β-hydroxybutyrate signaling may alter intracellular gene expression (e.g., via histone deacetylase [HDAC] inhibition; 116) which may impact cellular metabolism (116, 127). In addition, the neuroprotective effects mediated by microglia may be activated by both receptor activation (125) and altered cellular metabolism (i.e., using ketones instead of glucose; 128). Moreover, β-hydroxybutyrate’s effects on the GABA-glutamate ratio are impacted by signaling mechanisms and metabolic alterations. Specifically, β-hydroxybutyrate-induced downregulation of HDAC signaling (129), together with the promotion of anaplerosis (130), may lead to an increase of GABA relative to glutamate (129–131).

In summary, through signaling and metabolic pathways, KT-induced β-hydroxybutyrate increases may attenuate pro-inflammatory cytokines, reactive oxygen species, and glutamate (vs. GABA), while regulating BDNF levels, and enhancing ATP and GABA (vs. glutamate). Given that neuroinflammation, oxidative stress, neuronal hyperexcitability, BDNF dysregulation, and brain energy deficits are implicated in PTSD pathophysiology (11, 25, 69–71, 113, 114), the inverse modulation of these pathways may underlie the therapeutic potential of KT in PTSD. The reviewed studies not only addressed ketone-related metabolic and signaling properties with broad physiological relevance but also specified PTSD-associated brain regions in which these effects may occur.

#### 4.4.2 Potential downstream effects on PTSD psychopathology

Integrating existing evidence, we propose a model in which KT-induced physiological alterations in specific brain regions may reduce PTSD symptomatology. Given the complexity of potential interactions between and within all stages of downstream effects, a comprehensive depiction in one figure could hinder structured visualization. Therefore, we separated downstream effects into three stages, while drawing on the literature to explain potential key interactions in the text. **Figure 3** illustrates a collection of possible effects by highlighting supported KT-induced neurophysiological alterations (Stage 1), their potential subsequent functional impact (Stage 2), and expected corresponding PTSD symptom-level effects (Stage 3).

Gary et al. (70) reported the mobilization of β-hydroxybutyrate in the PFC under acute stress to account for energy shortages through increased ATP production. El Karkafi et al. (69) further described how stress-related reactive oxygen species may negatively impact the functioning of the PFC. Further, deficits in GABA within the PFC have been linked to cognitive impairments and disrupted emotion regulation in chronically stressed individuals (132). By increasing prefrontal ATP and GABA availability, and reducing reactive oxygen species and pro-inflammatory cytokines, KT may enhance PFC functionality (69, 70, 132–134). Enhanced prefrontal functioning could promote better regulation of intrusive memories and hyperarousal, as well as mitigation of avoidance behavior (133, 135, 136) - changes that could result in improved mood and subsequent reductions in both hyperarousal and intrusions (137, 138). Prefrontal inflammation has likewise been shown to interfere with the PFC’s top-down regulatory control over the amygdala (134). For PTSD, dysregulated PFC-amygdala connectivity represents a well-established neural correlate (133). Enhancing prefrontal functioning may therefore facilitate the downregulation of amygdala activity, potentially reducing exaggerated emotional and fear responses (139).

Exposure to traumatic and chronic stress have been linked to inflammation and oxidative stress within the amygdala (140–142). Moreover, findings from animal models of PTSD suggest that trauma- and stress-related experiences may be accompanied by dysregulation of amygdala BDNF signaling (143, 144). In individuals with PTSD, such alterations could plausibly contribute to amygdala hyperactivity and altered amygdala-dependent learning, which may increase fear responses and sensitivity to trauma-related stimuli (118, 145–148). These pathophysiological factors may promote hyperarousal and intrusive symptoms, impair emotional regulation, and foster avoidance behavior (149–151). KT-induced reductions in reactive oxygen species and pro-inflammatory cytokines, together with improved BDNF regulation, and enhanced GABA, may counteract oversensitivity of the amygdala and consequently yield improvements in PTSD symptomatology (118, 150, 152, 153).

The hippocampus, also identified as relevant in the reviewed preclinical PTSD literature (73), contributes importantly to memory contextualization, processing, consolidation (154–156), and stress regulation (157). This stress-regulatory role operates primarily through the attenuation of the HPA axis (157, 158). In PTSD, hippocampal dysfunction may be driven by converging pathophysiological processes, including elevated reactive oxygen species, increased pro-inflammatory cytokine activity, reduced BDNF signaling, disrupted GABA-glutamate balance, and cellular damage. These factors may impair hippocampal function and contribute to smaller hippocampal volumes, as reported in PTSD populations (114, 159–162).

Tanelian et al. (73) reported that β-hydroxybutyrate downregulates pro-inflammatory cytokines in the ventral hippocampus in a rodent model of PTSD. Additional KT-induced effects, such as increased hippocampal GABA levels (163), reduced reactive oxygen species (164), and increasing hippocampal BDNF expression (140), may support hippocampal health and potentially reverse metabolic impairments (165–167). Such neuroprotective effects may translate into improved stress regulation and memory function (157, 158, 168), which may, in turn, contribute to the mitigation of intrusive symptoms and hyperarousal in PTSD.

El Karkafi et al. (69) also described how stress-related reactive oxygen species may negatively affect the functioning of the insula. Low GABA concentrations in the insula have likewise been linked to PTSD (169). The insula is involved in interoceptive awareness and integrates signals related to sympathetic activation and affective states (170, 171). PTSD features such as dissociation, hyperarousal, and impaired emotion regulation may be partly linked to insular dysfunction, given the insula’s connectivity with the amygdala and the PFC (133, 135, 136, 172). Attenuating PTSD-associated insular levels of reactive oxygen species and enhancing insular GABA through KT may therefore contribute to significant PTSD symptom relief.

Another region of note is the cerebellum (72). Preston et al. (72) observed increased oxidative stress, GABA mobilization, and metabolic alterations consistent with an energy deficit in the cerebellum of rodent models vulnerable to developing PTSD-like symptomatology. Beyond its classical role in motor control, the cerebellum contributes to emotional processing, memory function, and stress responsivity (173–177). By reducing reactive oxygen species and increasing ATP and GABA levels, KT may support cerebellar functional integrity in circuits supporting affective processing, stress responsivity, and memory integration (173–177). Such neurobiological restoration could plausibly translate to attenuated hyperarousal and less intrusive re-experiencing, as well as contribute to overall mood stabilization.

These potential downstream effects are not intended to be exhaustive, but rather an illustration of how physiological changes described in the reviewed studies (i.e., increased GABA and ATP, improved BDNF regulation, and reduced levels of reactive oxygen species and pro-inflammatory cytokines) may plausibly translate into specific PTSD symptom-level effects, thereby offering a conceptual link between neurobiological alterations, functioning, and clinical manifestations.

### 4.5 Limitations

While being the first of its kind, this review should be regarded in light of its limitations. Importantly, the evidence base is small and dominated by narrative reviews, preclinical, case, and pilot studies. This limitation restricts the strength and generalizability of the findings. Furthermore, reliance on publicly available outcomes raises the possibility of publication bias, and despite a comprehensive search across several databases and registers, relevant unpublished reports may have been missed.

While the inclusion of different forms of KT strengthens the comprehensiveness of the current review, these forms may differ in their metabolic impact, adherence requirements, and safety profiles, making comparisons particularly challenging. Furthermore, we did not include studies that examined the effects of fasting or mere caloric restriction. Our rationale was that although such approaches may promote ketogenesis, they may be less sustainable across extended periods. Another limiting factor is the interpretation of findings on acetate supplementation in the context of KT. We considered this approach because acetate supplementation resulted in four times higher serum ketone levels in stressed animals, which meets our working definition for KT and appeared relevant in the context of this study.

A further methodological limitation concerns the moderate inter-rater agreement between the two independent reviewers during screening and eligibility assessment. Initial discrepancies were largely attributable to the broad search strategy and inclusion criteria, which were designed to capture evidence across heterogenous study designs. During screening, both reviewers were instructed to evaluate whether records focused on KT and PTSD or its core symptomatology. However, the concept of “focus” was initially applied differently. One reviewer adopted a broader interpretation, including records that addressed mechanisms which might be generally relevant to metabolism and post-traumatic stress, even where the intersection between KT and PTSD-specific symptomatology was absent. Because this review aimed to synthesize evidence specifically linking KT with PTSD-related outcomes rather than to review both domains separately, all initial discrepancies were resolved by assessing whether the full text established such a link. The small number of potentially eligible records after screening made Cohen’s Kappa particularly sensitive to initial disagreements in the eligibility phase. In this phase, several borderline cases were discussed. For example, we discussed one study that focused on a patient with bipolar disorder, who was not explicitly diagnosed with PTSD. However, this study reported PTSD symptoms before and after treatment, with the patient’s initial score being consistent (i.e., threshold exceedance) with a diagnosis of PTSD.

In other cases, the relationship between KT and PTSD was discussed conceptually rather than forming the primary intervention target or outcome focus. Initial disagreements were resolved by consensus after evaluating whether studies provided a defensible connection between KT and PTSD-related symptomatology and whether they reported outcomes relevant to safety, feasibility, or efficacy. Importantly, all discrepancies were resolved unanimously, and the discussions helped refine the operational application of the inclusion criteria.

Another limitation is that two of the included articles may report different analyses from the same pilot trial. Both Youssef et al. (86) and Locklin et al. (65) report a 6-week ketone supplementation study with 14 completers recruited at Augusta University, with one overlap in authorship. Although these patterns suggest that the reports may derive from the same pilot trial, inconsistencies between the articles preclude definitive conclusions. Notably, Locklin et al. (65) reported equal allocation between the intervention and control groups, whereas Youssef et al. (86) described eight participants in the intervention group and 6 in the control group. Furthermore, the description of the recruitment strategy is not identical between both articles. Given that the two articles address distinct outcomes (i.e., physiological safety markers in one article and PTSD symptoms in the other one), we treated them as separate reports in the analysis, while noting the possibility that they derive from the same participant cohort.

Our mechanistic modelling is limited. It should be best interpreted as conceptual and hypothesis-generating. In addition, it is not exhaustive in the sense that some relevant brain regions (e.g., anterior cingulate cortex, striatum, locus coeruleus) and mechanistic interactions (e.g., between GABA and reactive oxygen species) have not been explicitly addressed. Mechanistic depictions reflect abstractions built on the reviewed literature and are therefore limited in comprehensiveness. Lastly, all human studies to date have enrolled very small cohorts without robust control groups, and no large-scale, well-controlled, randomized trials or naturalistic studies have yet been published. This leaves the preliminary efficacy, feasibility, and safety analysis underpowered and susceptible to random variation and other phenomena.

### 4.6 Research gaps and recommendations

Despite growing research on KT for PTSD during the past five years, we identified numerous research gaps. First, we found that mechanistic and preclinical evidence remains limited, i.e., only a few narrative reviews and rodent studies addressed the potential of KT for PTSD. We recommend researchers further test the identified mechanistic pathways through which KT may attenuate PTSD pathophysiology. In addition, clinical studies in PTSD populations are scarce. As a result, high-level human efficacy and feasibility data are absent. We therefore recommend region-specific feasibility studies, followed by powered, long-term randomized controlled trials. For clinical trials, we recommend the application of a wide range of recruitment strategies and minimizing participant burden where possible (e.g., concise assessments). Moreover, the initial two-week adaptation phase to the ketogenic diet should be treated as a distinct period by researchers and clinicians. Participants should be informed in advance about potential adverse effects during this phase to reduce early dropouts. Given inter-individual variability in responses to KT, safety markers (e.g., lipids, inflammatory markers, carnitine levels, liver enzymes) should be monitored individually. Finally, research should explore KT as an adjunct to trauma-focused psychotherapy in controlled settings, to determine whether metabolic modulation can enhance or accelerate psychotherapeutic effects.

### 4.7 Conclusion

KT may be a promising physiological approach to the treatment of PTSD. Evidence-supported mechanistic pathways, preliminary efficacy in animal models, and data from observational studies suggest its potential. However, large randomized controlled clinical trials and well-designed naturalistic studies are still lacking, and they are needed to investigate clinical efficacy. In the absence of reported PTSD-specific safety signals, we recommend carefully testing different forms of KT (e.g., ketogenic diets, exogenous ketones, ketone precursor supplementation, and combined approaches) in larger cohorts of individuals with PTSD. Overall, the data suggest that KT should be further examined as both a standalone treatment and adjunct to established trauma-focused psychotherapy.

## Footnotes

1 Specifically, GLUT1 is necessary for the transport of glucose into the brain (76), whereas GLUT3 transports glucose into neurons (77). In addition, GLUT4 is a central player in brain insulin resistance and hippocampal memory processing (78). Turkson et al. (71) mention just GLUT generally.

2 Brain regions, central in the pathophysiology of PTSD (81, 82).

3 These alterations are commonly observed in individuals with PTSD (Hayes, 83, 84).

4 Glutamate enhances neuron excitability, while GABA depicts calming effects (85).
