# Supplementary material for: Ketogenic therapy as a potential novel treatment approach for PTSD: an integrative review and proposed mechanistic model
Source: Front Psychiatry. 2026 Sep 9;17:1854499. doi: 10.3389/fpsyt.2026.1854499 (PMC13598748; doi:10.3389/fpsyt.2026.1854499)
Supplement: Supplementary file 1 [file SupplementaryFile1.docx]

**Ketogenic Therapy as a Potential Novel Treatment Approach for PTSD:**

**An Integrative Review and Proposed Mechanistic Model.**

**Supplemental Material**

**Flow Diagram Initial Search**

Figure A. *PRISMA Flow Diagram of the Initial Search Process.*

Records removed *before screening*:

Duplicate records removed

(n = 36)

Records identified from:

Databases (n = 112)

Registers (n = 16)

**Identification**

Records screened

(n = 92)

Records excluded

(n = 72)

Reports sought for retrieval

(n = 20)

Reports not retrieved

(n = 2)

**Screening**

Reports assessed for eligibility

(n = 18)

Reports excluded:

Irrelevant outcome (n = 7)

Studies included in review

(n = 11)

**Included**

Source: Page MJ, et al. BMJ 2021;372:n71. doi: 10.1136/bmj.n71.

This work is licensed under CC BY 4.0. To view a copy of this license, visit <https://creativecommons.org/licenses/by/4.0/>

*Note:* The initial inter-rater agreement at the screening stage was (Cohen’s κ = .50). Initial inter-rater agreement for the eligibility assessment was Cohen’s κ = .44).

**Flow Diagram Search Update**

Figure B. *PRISMA Flow Diagram of the Updated Search Process.*

Records removed *before screening*:

Duplicate records removed

(n = 15)

Records identified from:

Databases (n = 24)

Registers (n = 8)

**Identification**

Records screened

(n = 17)

Records excluded

(n = 8)

Reports sought for retrieval

(n = 9)

Reports not retrieved

(n = 0)

**Screening**

Reports assessed for eligibility

(n = 9)

Reports excluded:

Irrelevant outcome (n = 4)

Studies included in review

(n = 5)

**Included**

Source: Page MJ, et al. BMJ 2021;372:n71. doi: 10.1136/bmj.n71.

This work is licensed under CC BY 4.0. To view a copy of this license, visit <https://creativecommons.org/licenses/by/4.0/>

*Note*: The inter-rater agreement at the screening stage was (Cohen’s κ = .48). Inter-rater agreement for the eligibility assessment was Cohen’s κ = .55).

| Table A. *Study Quality Hierarchy: Ranking Level of Evidence by Study Design.* | |  |
| --- | --- | --- |
|  |  |  |
| **High Quality Evidence** | |  |
| 1 | Systematic reviews (SRs) / meta-analyses (MAs) of controlled trials |  |
| 2 | Randomized controlled trials (RCTs) |  |
|  |  |  |
| **Moderate Quality Evidence** | |  |
| 3 | Non-randomized controlled trials |  |
| 4 | SRs / MAs of observational studies |  |
| 5 | Case control studies |  |
|  |  |  |
| **Low Quality Evidence** | |  |
| 6 | Uncontrolled trials |  |
| 7 | Case series / case reports |  |
| 8 | SRs / MAs of preclinical / mechanistic studies |  |
| 9 | Preclinical / mechanistic studies |  |
| 10 | Unsystematic reviews |  |
|  |  |  |
|  |  |  |
| *Note: This hierarchy is based on principles from George et al. (2021) and Guyat et al. (2008) and adjusted to the methodology of this integrative review. Certainty of evidence is discussed separately.* | |  |
|  |  |  |
|  |  |  |
|  |  |  |

| Table B. *Included Studies and Methodological Quality Assessment ordered by Study Type.* | | | | | | |
| --- | --- | --- | --- | --- | --- | --- |
|  |  |  |  |  |  |  |
| Number | Authors | Title | Category | Specification | Level of Evidence^1^ | Methodological Quality^2^ |
|  |  |  |  |  |  |  |
| 1 | El Karkafi et al. (2023) | Ketogenic Diet and Inflammation: Implications for Mood and Anxiety Disorders. | Unsystematic Review | Mechanistic | low | moderate |
| 2 | Gary et al. (2024) | Exploring metabolomic dynamics in acute stress disorder: amino acids, lipids, and carbohydrates. | Unsystematic Review | Mechanistic | low | high |
| 3 | Turkson et al. (2019) | Neuroendocrine Drivers of Risk and Resilience: The Influence of Metabolism & Mitochondria | Unsystematic Review | Mechanistic | low | moderate |
| 4 | Preston et al. (2021) | Cerebellar and multi-system metabolic reprogramming associated with trauma exposure and post-traumatic stress disorder (PTSD)-like behavior in mice | Mechanistic | Animal | low | high |
| 5 | Tanelian et al. (2023) | Effect of acetate supplementation on traumatic stress-induced behavioral impairments in male rats. | Preclinical | Animal | low | high |
| 6 | Yamanashi et al. (2020) | Beta-hydroxybutyrate, an endogenous NLRP3 inflammasome inhibitor, attenuates anxiety-related behavior in a rodent post-traumatic stress disorder model. | Preclinical | Animal | low | high |
| 7 | Yoshioka et al. (2026) | Oral administration of MCT oil reduces anxiety like behavior and is associated with differences in serum BDNF responses in a rat model of PTSD. | Preclinical | Animal | low | high |
| 8 | Anekwe et al. (2020) | Ketogenic Diet-induced Elevated Cholesterol, Elevated Liver Enzymes and Potential Non-alcoholic Fatty Liver Disease. | Observational | Case study | low | moderate |
| 9 | Bellamy & Laurent (2025) | Transdiagnostic remission of psychiatric comorbidity in post-traumatic stress disorder, ADHD, and binge-eating disorder using ketogenic metabolic therapy: a retrospective case report. | Observational | Case study | low | high |
| 10 | Halabitska et al. (2026) | War, diet, and PTSD in Ukrainian youth. | Observational | Cross-sectional  study | moderate | high |
| 11 | Laurent (2024) | Retrospective case study: ketogenic metabolic therapy in the effective management of treatment-resistant depressive symptoms in bipolar disorder. | Observational | Case study | low | high |
| 12 | Laurent (2026) | Ketogenic metabolic therapy for treatment-resistant post-traumatic stress disorder (PTSD): a retrospective case report. | Observational | Case study | low | high |
| 13 | Laurent et al. (2025) | Ketogenic metabolic therapy for schizoaffective disorder: a retrospective case series of psychotic symptom remission and mood recovery. | Observational | Case series | low | high |
| 14 | Edwards et al. (2024) | Exploring diet-induced ketosis with exogenous ketone supplementation as a potential intervention in post-traumatic stress disorder: a feasibility study. | Clinical | Pilot trial | low | high |
| 15 | Locklin et al. (2024) | The Impact of Ketone Salt Supplementation on Blood Pressure and Health Parameters in Adults Diagnosed with PTSD – A Pilot Study. | Clinical | Pilot trial | high | high |
| 16 | Youssef et al. (2022) | A randomized, double-blind, clinical pilot trial of adjunct ketone supplement compared to placebo for treating posttraumatic stress disorder. | Clinical | Pilot trial | high | high |
|  |  |  |  |  |  |  |
| *Note: ^1^According to hierarchy of evidence ranked by study design, ^2^according to Critical Appraisal Tool criteria (CAT; Crowe et al., 2012). Inter-rater agreement (Cohen’s κ) was κ = 1 for the hierarchy of evidence ranking in both the initial search and the search update as well as κ = .74 for the initial and κ = 1 for the search update CAT assessment. Certainty of evidence including sample sizes is acknowledged in the manuscript text.* | | | | | | |

**References**

George, A., Kleinlugtenbelt, Y. V., & Madden, K. (2021). Hierarchy of Evidence and Common Study Designs. In M. Bhandari (Ed.), *Evidence‐Based Orthopedics* (1st ed., pp. 7–10). Wiley. https://doi.org/10.1002/9781119413936.ch2

Guyat, G., Drummond, R., Maureen O., M., & Deborah J., C. (2008). *Users’ Guides to the Medical Literature* (Second Edition). American Medical Association. DOI: 10.1036/0071590382
